# Use of proposed systolic and myocardial performance indices derived from simultaneous ECG and PCG recordings to assess cardiac function in healthy Beagles

**DOI:** 10.14202/vetworld.2022.1785-1797

**Published:** 2022-07-25

**Authors:** Karlo Romano B. Gicana, Chirutchaya Pinidmontree, Kitchanan Kosalathip, Siraphop Sirirut, Siripen Komolvanich, Sariya Asawakarn, Walasinee Sakcamduang, Phornphop Naiyanetr, Kittipong Tachampa

**Affiliations:** 1 The International Graduate Program of Veterinary Science and Technology, Faculty of Veterinary Science, Chulalongkorn University, Bangkok, Thailand; 2 Department of Veterinary Clinical Sciences, College of Veterinary Medicine, University of the Philippines Los Baños, Laguna, Philippines; 3 Department of Physiology, Faculty of Veterinary Science, Chulalongkorn University, Bangkok, Thailand; 4 Cardiovascular Engineering and Artificial Organs (CardioArt) Laboratory, Department of Biomedical Engineering Department, Mahidol University, Nakhon Pathom, Thailand; 5 Biomarkers in Animal Parasitology Research Group, Chulalongkorn University, Bangkok, Thailand; 6 Department of Clinical Sciences and Public Health, Faculty of Veterinary Science, Mahidol University, Nakhon Pathom, Thailand

**Keywords:** cardiac time intervals, dogs, electrocardiography, healthy, phonocardiography

## Abstract

**Background and Aim:**

Cardiac time intervals (CTIs) can provide important information on the electrical and mechanical properties of the heart. We hypothesized that cardiac function can be described using the combined power of electrocardiography (ECG) and phonocardiography (PCG) signals. This study aimed to (1) validate a novel custom device in measuring CTI parameters; (2) compare CTI parameters with a commercially available device and standard transthoracic echocardiography (STE); and (3) compare calculated systolic performance index (SPI) and myocardial performance index (MPI) with Tei index from the STE.

**Materials and Methods:**

This study determined CTIs based on simultaneous ECG and PCG recordings in 14 healthy Beagle dogs using the custom-built device. These CTI parameters were compared with a commercially available device (Eko DUO ECG + Digital Stethoscope; Eko DUO) and the STE. Agreement of CTI parameters between the custom device and the commercially available device or STE was evaluated. Calculated SPI and MPI based on Wigger’s diagram were proposed, compared with SPI and Tei index, and correlated with STE parameters.

**Results:**

We found that the ECG and PCG parameters measured from the custom-built device did not differ from the commercially available device and the STE. By combining ECG and PCG signals, we established CTI parameters in healthy dogs including indices for systolic function (SPI: QS1/S1S2) and global cardiac function {F1 ([QS1+S2]/S1S2), F2 ([RS1+S2]/S1S2), and F3 (RS1 + [QS2-QT]/S1S2)}. The SPI, F2, and F3 were comparable with echocardiographic parameters describing systolic (Pre-ejection period/left ventricular ejection time [LVET]) and Tei index ([MCOdur-LVET]/LVET), respectively. Only SPI and F3 were correlated significantly with MCOdur and heart rate, respectively.

**Conclusion:**

We have validated the use of the custom-built device to describe CTIs that are comparable to the commercially available device and STE in healthy Beagles. The proposed SPI and MPI derived from CTI parameters can be useful in clinical practice to describe the cardiac function, especially in areas where access to STE is constrained.

## Introduction

**Figure-1:**
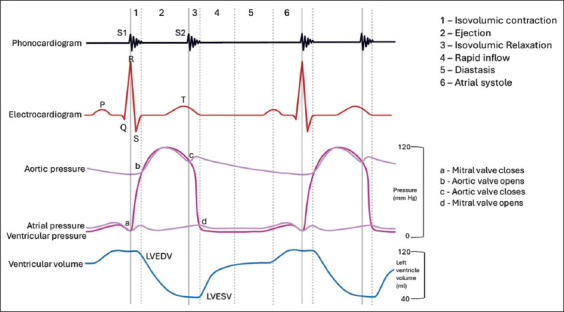
Cardiac time intervals and events described in the cardiac cycle. Image modified from Wigger’s diagram [1] (Artist: Bryan Paul I. Bulatao).

The heart is a dynamic organ and is characterized by simultaneous cardiac events happening within a cardiac cycle. Based on the Wiggers diagram (Figure-1) [1], the relationship between electrical and mechanical events of the heart can be described using cardiac time intervals (CTIs). The ability to use these non-invasive monitoring techniques to assess cardiac function is useful in clinical and research situations. The use of CTIs to detect heart failure was previously investigated in humans [2] and pigs [3]. In dogs with valvular diseases, changes in CTIs were observed [4, 5]. Among several CTI parameters, systolic time intervals (STIs) and myocardial performance index (MPI or Tei index) have gained interest due to their ease of recording and usefulness in determining left ventricular and global cardiac function. Conventionally, both parameters can be obtained using the STE. The STI can be measured using an M-mode or Doppler echocardiography through the systolic phase, including the interval from the QRS complex to the beginning of left ventricular (LV) ejection (Pre-ejection period; PEP) as well as the left ventricular ejection time (LVET) [6]. The ratio of both parameters (PEP/LVET) was further developed to minimize the heart rate (HR) dependency and was proposed as a sensitive indicator of systolic performance [7]. The STI, as a systolic performance index (SPI), has been extensively studied in human medicine [8–15] as compared to veterinary medicine. This may probably be due to available methods employing more advanced techniques to assess LV function. In contrast, MPI has been extensively used in both human and small animal medicine [5, 16–20]. The MPI derived from pulsed Doppler echocardiography is defined as the sum of isovolumetric contraction time and the isovolumetric relaxation time divided by the LVET [21–23]. The MPI has been reported to reflect global cardiac function (i.e., systolic and diastolic function in human patients) [24, 25]. In dogs, the LV MPI was reported to correlate with the severity of disease in mitral regurgitation, myxomatous mitral valve diseases (MMVD), and dilated cardiomyopathy (DCM) [4, 26].

Although physical examination and other diagnostic tools can distinguish asymptomatic and symptomatic patients in cases of cardiac diseases like MMVD [27], this information does not describe cardiac function. The use of parameters to describe cardiac function is important, especially in the transition to a more progressive stage of the disease. As of today, the gold standard tool to describe the cardiac function and diagnose cardiac diseases in dogs is echocardiography [22, 27]. However, limited access to this machine makes it very challenging and costly for clinicians and owners. Therefore, it is imperative to find a new diagnostic tool to measure cardiac function that is accessible, accurate, cheap, and reliable. This can support existing diagnostic tools in describing cardiac function and the progression of the disease. Current technology paved the way for the development of commercial devices employing the use of phonocardiography (PCG) and electrocardiography (ECG) to derive CTI parameters in humans and animals [2, 28–30]. These CTI parameters include electromechanical activation (QS1) and electromechanical systole (QS2), left ventricular systolic time (S1S2), and left ventricular diastolic time (S2S1) [28, 29]. These were valuable in detecting left ventricular dysfunction in humans [31–33]. It can be used to evaluate systolic function and identify heart failure [2, 34, 35]. In animals, it can be used to evaluate both cardiac functions [30] and identify acute myocardial ischemia [3]. Based on Wiggers’ diagram (Figure-1), the SPI and MPI can be derived by combining ECG and PCG parameters. The SPI can be computed using QS1 duration divided by S1S2 duration. In addition, MPI can be calculated using three different equations as proposed by the authors: {([QS1+S2]/S1S2), ([RS1+S2]/S1S2), and (RS1 + [QS2-QT]/S1S2)} reflecting the Tei index from STE as proposed in this study. Theoretically, the use of CTIs may be applicable to dogs. However, there was limited literature available describing CTIs from simultaneous ECG and PCG in healthy dogs [36]. The existing data regarding CTIs were not fully elaborated, especially on their clinical use in dogs. Moreover, the available commercial device measuring both ECG and PCG device ([The Eko DUO ECG + Digital Stethoscope; Eko DUO] Eko Devices Inc., California) is designed mainly for humans with proprietary algorithms and software. Recently, we reported the use of the Eko DUO device to determine CTIs and its feasibility in measuring cardiac function in MMVD dogs [37]. Nevertheless, the development of a novel device designed primarily for dogs can be a supportive tool in describing and evaluating cardiac functions, particularly in areas where STE is limited or inaccessible. In addition, this will pave the way for the development of algorithms and software appropriate for clinical use in dogs.

This study aimed to use the simultaneous ECG and PCG recordings to determine CTIs and their utilization in determining global cardiac functions in healthy dogs. Specifically, this study aimed to (1) validate the use of a custom device in measuring CTIs; (2) compare the measured CTIs with the commercially available device and standard transthoracic echocardiographic parameters; and (3) compare the SPI and MPI from the custom device with PEP/LVET and Tei index from STE. These reference points on CTI parameters can potentially be used as a supportive diagnostic tool in describing cardiac function in dogs.

## Materials and Methods

### Ethical approval

The handling and use of animals in this study were done in strict compliance with the research protocol (Protocol no. 1931013) as reviewed and approved by the Institutional Animal Care and Use Committee, Faculty of Veterinary Science, Chulalongkorn University, Bangkok, Thailand.

### Study period and location

The study was conducted from January 2019 to June 2021 at Nakhon Pathom campus of Faculty of Veterinary Science, Chulalongkorn University.

### Animals

This study was conducted as per the Guide for the Care and Use of Laboratory Animals [38]. All methods were done as per the pertinent rules (ARRIVE Essential 10) [39] and followed guidelines for animal experimentation. Fourteen healthy Beagle dogs from the breeding colony of the Department of Obstetrics and Gynecology, Faculty of Veterinary Science, Chulalongkorn University, were used in this study. Dogs of the same breed, age, and weight were included in this study. These dogs were healthy based on physical, clinical, echocardiographic examinations, and cardiac biomarkers values (cTnI, NT-ProBNP, and 5HT). Dogs with arrhythmias, underlying disease, and who have undergone any medications were excluded from the study.

### Development of the custom-built device and its ECG/PCG signal recording

**Figure-2:**
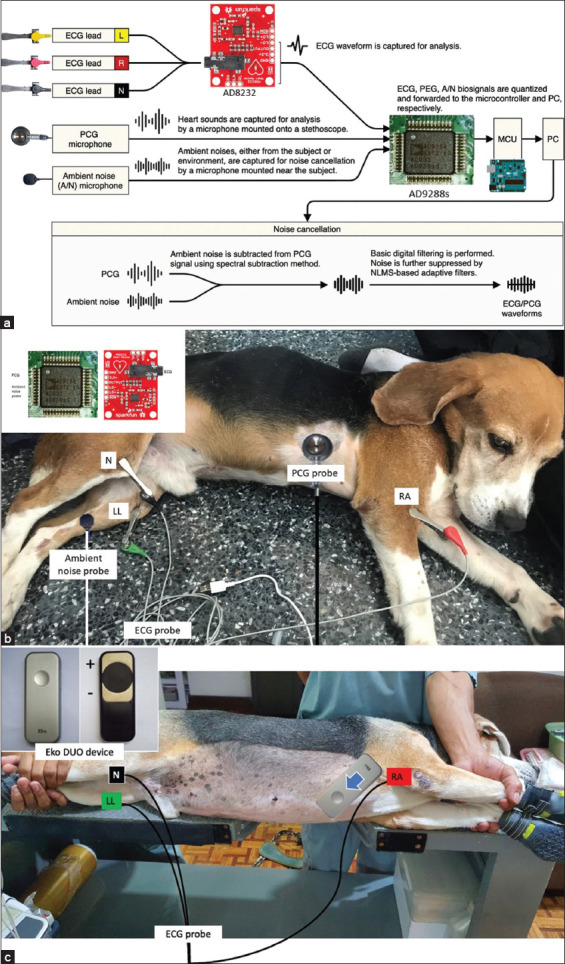
(a) The custom device recorded simultaneous ECG and PCG recordings through ECG lead cables, stethoscope, and ambient noise probe as demonstrated in a schematic diagram (b) and its attachment in a healthy dog through the right arm (RA) and left leg (LL). (c) The Eko DUO device was placed on the thoracic skin from the base to the apex of the heart direction. ECG=Electrocardiography and PCG=Phonocardiography.

The custom-built device is a custom ECG/PCG signal acquisition module. This was attached to the animal using its ECG lead cables, chest piece of a stethoscope, and ambient noise probe, as demonstrated in Figure-2a. Briefly, the ECG signals were received through ECG lead wires; then were amplified and quantified through the AD8232 analog front end (AFE) and AD9288 analog-to-digital converter (ADC), respectively. In addition, the signals were captured at a 100k Hz sampling rate with the same resolution of AFE and ADC. The signals were filtered using zero-phase digital filtering and adaptive noise suppression techniques to suppress 50 Hz harmonics (utility line interference) and background noises. Meanwhile, the PCG sensors utilized the commercially available Littmann^®^ Cardiology III™ Stethoscope chest piece (3M™, Minnesota, United States) coupled with a Boya BY-M1 microphone (Boya, Shenzhen, China). The device was powered using either a switched-mode power supply or an internal 10AH 4-cell lithium-ion battery.

**Figure-3:**
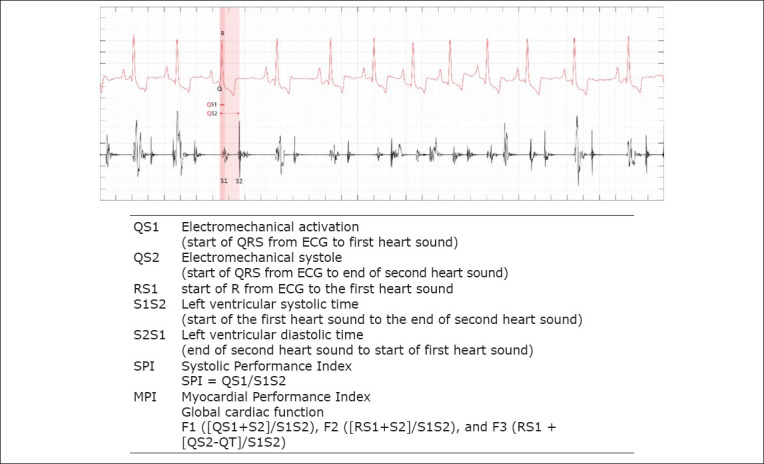
The cardiac time interval parameters (ms) and derived values represent systolic and global cardiac functions using simultaneous ECG and PCG. ECG=Electrocardiography and PCG=Phonocardiography.

Using AD8232, the different ECG waveforms were captured for analysis through the different ECG lead attachments in the animal, as shown in Figure-2a and b. These ECG recordings, together with the audio signals from the stethoscope (PCG) and the microphone, were collected by the AD9288s ADC to yield simultaneous ECG and PCG recording signals. These simultaneous recordings were processed and analyzed to disregard ambient noise using digital filters from a microcontroller and a computer. The processed ECG and PCG recording signals were subsequently analyzed and measured using Matlab® software (Mathworks Inc., Massachusetts, United States) and were reported in milliseconds (MS). These simultaneous ECG and PCG recordings were then further used for the measurement of CTI parameters, as described in Figure-3.

### Signal recording from Eko DUO ECG + digital stethoscope

The Eko DUO device (Eko Devices Inc., California, United States) was placed on the skin surface, following from the base to the apex direction of the heart. The animal was placed on left lateral recumbency on the echocardiography table, as shown in Figure-2c. ECG gel was applied on the skin surface to facilitate proper conduction and recording of signals. This Eko DUO device has positive and negative electrodes (Figure-2c; inset) with a 0.01 Hz high pass filter with a 50 Hz main filter and a 500 Hz sampling rate. The device recorded simultaneous ECG and PCG signals that were recorded and stored through Bluetooth^®^ 4.0 low-energy technology. The CTI parameters were briefly described in Figure-3 and were measured using proprietary algorithms and software through a cloud-based Eko App (Eko Devices Inc., California, United States). The CTI values were reported in ms and based on the average of 5 subsequent beats.

### The STE and echocardiographic parameters measurement

The STE was performed by an experienced veterinarian to provide consistent measurement and minimize variability. An ultrasound machine equipped with the 2–8 MHz transducer phased-array ultrasound probe (M9, Mindray^®^, Shenzhen, China) was used in this study. The probe was placed on the skin surface of both the left and right heart at the level of the 3^rd^ to 5^th^ intercostal spaces to obtain a standard right, left, and apical parasternal view of the heart. Echocardiographic parameters were measured, describing dimensions (left atrial to aortic root ratio [LA/Ao], left ventricular internal diameter during diastole normalized to body weight [LVIDDN], and left ventricular internal diameter during systole normalized to body weight [LVIDSN]). The cardiac function parameters Simpson’s ejection fraction (EF) and fractional shortening (FS), STIs including a PEP, LVET, diastolic time intervals including (MCOdur), and global (Tei index) time intervals were measured based on described and accepted standard views [39]. The cardiac dimensions were normalized based on their body weight and were computed based on Cornell’s equation [40]. The various CTI parameters were recorded and measured at the level of transmitral inflow and left ventricular outflow tracts (PEP, LVET, PEP/LVET, MCOdur, and Tei index). These parameters were compared with related CTI parameters from the Eko DUO and our custom-built device.

### Definitions and measurement of CTI parameters

The simultaneous ECG and PCG recordings were recorded using lead attachments and a stethoscope, as demonstrated in Figure-2. These recordings yielded CTI parameters including QS1, QS2, S2, S1S2, and S2S1 as shown in Figure-3. Using these CTI parameters, systolic and global cardiac function indices can be computed using the novel formula for SPI and MPI as this study proposed: SPI (QS1/S1S2), MPI-F1 (QS1+S2)/S1S2, MPI-F2 (RS1+S2)/S1S2, and MPI-F3 (RS1 + [QS2-QT]/S1S2).

### Cardiac biomarkers

A 5 mL of blood was collected from each animal and was centrifuged for 15 min at 100× *g*, 2–8°C to collect plasma. The cardiac biomarkers were measured based on quantitative sandwich ELISA (NT-pro BNP and Troponin-I) and competitive ELISA (serotonin) using plasma. The tests were species-specific and were done based on the procedure described and recommended in the manufacturer’s protocol (MyBioSource Inc., United States). The cardiac biomarker values were reported in mean ± standard deviation (SD).

### Statistical analysis

The clinical descriptive values of healthy dogs, including echocardiographic parameters and cardiac biomarkers were reported as mean ± SD. The ECG, PCG, and CTI parameters were reported in mean ± SEM and statistically analyzed using commercially available software. These parameters from STE, Eko DUO, and the custom device were tested for normality using the Kolmogorov–Smirnov test. These were compared between and among groups using unpaired t-test, ANOVA, and *post hoc* Tukey’s test at p < 0.05, respectively. The Bland-Altman test was used to evaluate for bias and agreement between the mean differences of values of the custom-built device and Eko DUO, as well as the custom-built device and STE. The relationship of CTI parameters between the STE and custom device was determined using the Pearson correlation test. The values were reported at a 95% confidence interval.

## Results

### Clinical description of the healthy dogs

A total of 14 healthy Beagle dogs were used in this study to measure, compare, and establish CTIs in healthy dogs. These dogs are clinically described as having a mean HR of 101 bpm, a mean body weight of 12 kg, and a mean age of 4 years. The dogs were subjected to STE examination describing the cardiac dimension, function, and CTIs based on standard measurements. The echocardiographic measurements are listed in Table-1 [5, 26, 40–44]. Parameters of cardiac dimension and function, including LA/Ao, LVIDDN, LVIDSN, EF, and FS were within normal limits and reflected the healthy status of these animals. The CTI parameters such as PEP, LVET, PEP/LVET, MCOdur, and Tei index were also all within normal limits. To further confirm the healthy heart of these dogs, the cardiac biomarkers of myocardial injury (cTnI and serotonin) and heart failure (NT-ProBNP) were tested. All cardiac biomarker values were also within the normal range, as shown in Table-1.

**Table 1:** Clinical description of healthy dogs in this study.

Parameters	Mean ± SD (n = 14)	Reference values
Canine patient characteristics		
Age (years)	4.39 ± 3.15	
Weight (kg)	12.28 ± 1.63	11.1 ± 10.30 [5]
HR (bpm)	101.57 ± 23.37	114 ± 24.00 [5]
Echocardiographic variables		
Dimension (cm)		
LA/Ao	1.45 ± 0.25	1.26 ± 0.13 [5]
LVIDDN**	1.50 ± 0.09	1.4 ± 0.18 [41]
LVIDSN**	0.93 ± 0.10	0.80 ± 0.17 [41]
Cardiac function (%)		
EF	54.06 ± 20.40	66.5 ± 6.4 [42]
FS	39.69 ± 17.72	39 ± 7.00 [5]
Systolic time interval (ms)		
PEP	33.00 ± 26.18	43.6 ± 8.70 [42]
LVET	170.55 ± 18.38	171 ± 20.00 [5]
PEP/LVET	0.20 ± 0.16	0.267 ± 0.055 [42]
Diastolic time interval (ms)		
MCOdur	239.65 ± 34.19	268 ± 29 [26]
Global		
Tei	0.42 ± 0.22	0.38 ± 0.10 [5]
Cardiac biomarkers		
cTNI (ng/mL)	0.0002 ± 0.0001	0.02 ± 0.01 [43]
NT pro-BNP (pmol/L)***	91.05 ± 31.93	435 ± 195.00 [43]
Serotonin (ng/mL)	30.41 ± 17.97	51.8 (SE 5.7) [44]

*Data were presented as mean ± SD. HR = Heart rate, LA/Ao = Left atrium, LVIDDN = Left ventricular internal diameter during diastole, normalized, LVIDSN = Left ventricular internal diameter during systole, EF = Ejection fraction, FS = Fractional shortening, SV = Stroke volume, PEP = Pre-ejection period, LVET = Left ventricular ejection time, MCOdur = Mitral valve close to open duration, Tei = Myocardial performance index using standard echocardiography, MCOdur-LVET/LVET, SE = Standard error.

** Reference values were normalized based on body weight [40].

*** Canine NT pro-BNP was measured using high sensitivity ELISA with a detection range of 36.89–2364.90 pmol/L. SD = Standard deviation

### Validation of the custom device by comparing with the Eko DUO device and STE

To validate the use of the developed device, nine healthy Beagle dogs with a mean body weight of 12 kg and a mean age of 6 years old were used to compare different ECG and PCG parameters, as shown in Table-2. The ECG and PCG values were not seen as significantly different among custom devices, Eko DUO, and STE.

**Table 2:** ECG and PCG parameters were measured compared among Eko DUO, standard transthoracic echocardiography, and the custom device.

Parameters	Eko DUO	Echo	Custom device	p-value
ECG (ms)				
PR interval	108.90 ± 4.08	111.10 ± 2.20	117.00 ± 8.92	0.60
QRS	61.33 ± 2.17	54.86 ± 2.74	55.87 ± 2.92	0.19
QT interval	212.60 ± 6.09	218.30 ± 11.07	207.20 ± 11.06	0.72
RR interval	568.90 ± 31.26	558.10 ± 34.86	528.20 ± 26.93	0.64
PCG (ms)				
S1S2	251.30 ± 17.72	-	235.60 ± 10.54	0.46
S2S1	274.30 ± 35.28	-	276.20 ± 18.36	0.96
S2 duration	101.30 ± 11.16	-	89.34 ± 8.96	0.41

The values were reported in mean ± SEM. ECG values were compared among Eko DUO, Echo, and a custom device using one-way ANOVA and *post hoc* Tukey’s at p < 0.05. PCG values were compared between Eko DUO and a custom device using an unpaired t-test at p < 0.05. ECG = Electrocardiography and PCG = Phonocardiography

### Establishment of CTIs using the custom-built device

Since the ECG and PCG parameters did not vary among devices, we utilized these combined parameters to describe CTIs using the custom device. The ECG parameters (i.e., PR, QRS, QT, and RR interval) were measured simultaneously with PCG parameters (i.e., S1S2 and S2S1 duration), as reported in Table-3. The electromechanical activation and systolic time were measured using QS1 and QS2, respectively. Using QS1 and S1S2 parameters, the SPI was calculated. Meanwhile, as a measure of global cardiac function, we computed three MPI formulas using the proposed formula from simultaneous ECG and PCG recordings from the custom device. The calculated MPI parameters of F1, F2, and F3 are shown in Table-3.

**Table 3:** CTI parameters were measured from the custom device using simultaneous ECG and PCG recordings in healthy dogs (n = 14).

CTI parameters (ECG+PCG)	CTI values (ms)
QS1	65.66 ± 22.82
QS2	299.50 ± 53.68
SPI	0.27 ± 0.02
RS1	37.74 ± 5.34
F1	0.55 ± 0.05
F2	0.43 ± 0.08
F3	0.54 ± 0.05

The values were reported in mean ± SD values. SPI=QS1/S1S2; MPI: F1 ([QS1+S2]/S1S2), F2 ([RS1+S2]/ S1S2), and F3 (RS1 + [QS2-QT]/S1S2) using custom device. ECG=Electrocardiography, PCG=Phonocardiography, CTIs=Cardiac time intervals, SD=*Standard deviation*

### SPI and MPI from the custom device compared with the Eko DUO and STE

**Figure-4:**
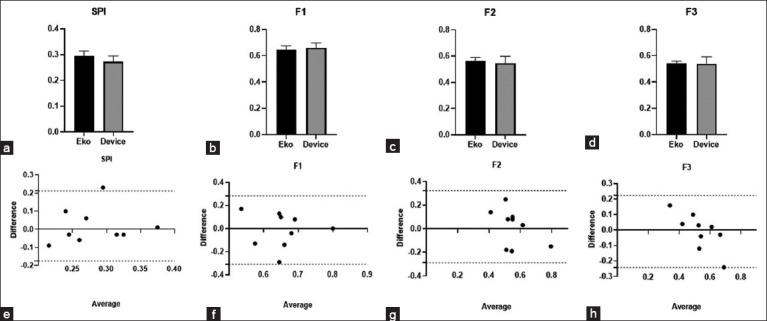
(a) Comparison of SPI, (b) MPI-F1, (c) MPI-F2, (d) MPI-F3 values (mean ± SEM) between Eko DUO and the custom device in healthy dogs using paired t-test at p < 0.05, and (e-h) The agreement between these parameters of the two devices was tested using the Bland Altman test at p < 0.05. The horizontal dash lines indicate 95% CI. SPI=QS1/S1S2; MPI: F1 ([QS1+S2]/S1S2), F2 ([RS1+S2]/S1S2), and F3 (RS1 + [QS2-QT]/S1S2). MPI=Myocardial performance index, SPI=systolic performance index.

We compared both SPI and MPI formulas from commercially available Eko DUO devices and our custom device as graphically represented in Figure-4. The QS1/S1S2 (SPI = 0.27 ± 0.07) from the device did not differ from the SPI from EkoDUO (0.29 ± 0.07) (p = 0.47) (Figure-4a). When compared the calculated MPI from the device (F1 = 0.66 ± 0.11, F2 = 0.55 ± 0.16, and F3 = 0.54 ± 0.16) and Eko DUO (F1 = 0.64 ± 0.12, F2 = 0.56 ± 0.10, and F3 = 0.54 ± 0.07) were found similar, (Figure 4b-d). In Figures 4e-h, the mean difference of the SPI (0.02, −0.18–0.21), F1 (−0.01, −0.31–0.28), F2 (0.02, −0.29–0.32), and F3 (−0.01, −0.24–0.22) was compared and revealed a close agreement between the two devices using Bland-Altman test.

**Figure-5:**
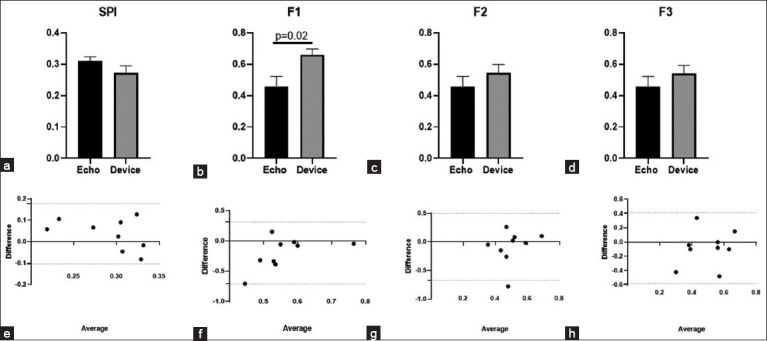
(a) Comparison of SPI, (b) MPI-F1, (c) MPI-F2, (d) MPI-F3 values (mean ± SEM) between standard transthoracic echocardiography (Echo) and the custom device in healthy dogs using paired t-test at p < 0.05, and (e-h) The agreement between these parameters of the two devices was tested using the Bland Altman test at p < 0.05. The horizontal dash lines indicate 95% CI. SPI=QS1/S1S2; MPI: F1 ([QS1+S2]/S1S2), F2 ([RS1+S2]/S1S2), and F3 (RS1 + [QS2-QT]/S1S2). MPI=Myocardial performance index, SPI=Systolic performance index.

Similarly, the SPI and MPI were compared between STE and the custom device, as shown in Figure-5. The PEP/LVET (0.31 ± 0.04) did not differ significantly with SPI (QS1/S1S2) from the device (0.27 ± 0.07), as shown in Figure-5a. The Tei index (0.46 ± 0.19) was significantly lower when compared with MPI-F1 (0.66 ± 0.11). In contrast, no observed differences were seen when the Tei index was compared to MPI-F2 (0.55 ± 0.16) and MPI-F3 (0.54 ± 0.16) from the device. In Figure-5e-h, the mean difference of the SPI (0.04, −0.10–0.18), F2 (−0.09, −0.67–0.50), and F3 (−0.08, −0.58–0.42) revealed a close agreement between the device and STE as compared using Bland-Altman test. In contrast, F1 demonstrated a wider bias range (−0.20, −0.71–0.31).

We continued to investigate further to find whether both SPI and the calculated MPI formulas correlate with the STE parameters, as demonstrated in Table-4. The SPI, F1, and F2 did not correlate with HR. In contrast, only F3 demonstrated a strong correlation with HR. The SPI did not correlate with cardiac dimension parameters, including LA/Ao, LVIDDN, and LVIDSN. Similarly, F1, F2, and F3 did not correlate with LA/AO, LVIDDN, and LVIDSN, respectively. EF and FS did not show any relationship with SPI, F1, F2, and F3, as shown in Table 4. As a measure of STI, SPI did not demonstrate a significant relationship with PEP, LVET, and PEP/LVET. The F1, F2, and F3 have similar observations with PEP, LVET, and PEP/LVET, respectively. As a measure of diastolic function, the MV E/A was also not seen as significantly correlated with the SPI, F1, F2, and F3. In the same observation, F1, F2, and F3 reported a non-significant relationship with MCOdur. In contrast, SPI has shown a moderate correlation with MCOdur. The global cardiac function parameter (Tei index) was not seen correlated with SPI, F1, F2, and F3.

**Table 4:** Relationship of CTIs from the custom device with STE parameters.

STE parameters	SPI	F1	F2	F3
HR	−0.06, 0.89	−0.38, 0.31	−0.45, 0.23	−0.76, 0.02*
Dimension				
LA/Ao	0.05, 0.90	−0.16, 0.68	0.16, 0.68	0.27, 0.49
LVIDDN	0.08, 0.83	0.23, 0.55	0.40, 0.28	0.54, 0.14
LVIDSN	−0.28, 0.46	−0.24, 0.53	0.01, 0.97	−0.44, 0.23
Cardiac function				
EF (Simpson)	−0.36, 0.35	−0.06, 0.88	0.30, 0.43	−0.42, 0.25
FS	0.27, 0.48	0.28, 0.46	0.16, 0.68	0.59, 0.09
Systolic time interval				
PEP	0.35, 0.36	0.05, 0.90	−0.12, 0.77	0.37, 0.33
LVET	0.25, 0.52	0.46, 0.21	0.22, 0.57	0.34, 0.37
PEP/LVET	0.16, 0.69	−0.32, 0.40	−0.28, 0.47	0.15, 0.70
Diastolic time interval				
MV E/A	−0.14, 0.71	0.41, 0.27	0.31, 0.42	0.22, 0.57
MCOdur	0.69, 0.04*	0.05, 0.91	−0.35, 0.36	0.50. 0.17
Global				
Tei	0.23, 0.56	−0.41, 0.28	−0.43, 0.24	−0.003, 0.99

The values were reported in r and *P* values, respectively.

* Indicates a significant difference at p < 0.05 using the Pearson correlation test. SPI=PEP/LVET from STE, SPI=QS1/S1S2 from a custom device, Tei index=MCO-LVET/LVET from STE. MPI: F1 ([QS1+S2]/S1S2), F2 ([RS1+S2]/S1S2), and F3 (RS1 + [QS2-QT]/S1S2) using a custom device, STE=Standard transthoracic echocardiography, HR=Heart rate, LA/Ao=Left atrium, LVIDDN=Left ventricular internal diameter during diastole, normalized, LVIDSN=Left ventricular internal diameter during systole, EF=Ejection fraction, FS=Fractional shortening, SV=Stroke volume, PEP=Pre-ejection period, LVET=Left ventricular ejection time, MCOdur=Mitral valve close to open duration, Tei=Myocardial performance index using standard echocardiography, CTIs=Cardiac time intervals

## Discussion

In this study, a simple and cheap prototype device was constructed from the ECG sensor, a stethoscope chest piece, and a microphone and was used to describe various CTI parameters in 14 healthy beagles. This prototype device was validated by comparing these CTI parameters with the CTIs obtained from the commercially available device and with the STE. We have successfully demonstrated that ECG and PCG parameters from our custom device did not differ from both the Eko DUO device and the STE with similar parameters. In addition, we have seen that CTI parameters from our device were comparable and repeatable with the commercial device. Remarkably, these CTI parameters displayed close agreement in measuring QS1/S1S2 (SPI), F1 ([QS1+S2]S1S2), F2 ([RS1+S2]/S1S2), and F3 (RS1 + [QS2-QT]/S1S2). We have shown that SPI and the calculated MPI-F2 and MPI-F3 formula from our device were similar to echocardiographic parameters describing systolic (PEP/LVET) and global cardiac function parameter (Tei index). Therefore, we have successfully validated our device in establishing CTIs to describe systolic, and global cardiac functions and proved the use of SPI and MPI to measure cardiac functions in healthy dogs.

The main advantage of this developed device was portability, as this can acquire and record simultaneous ECG and PCG recordings using a single device. This allowed the analysis of the proposed novel indices (SPI and MPI), therefore, making it convenient for clinical use. However, the use of this developed device is limited to the 20–100 kHz PCG sensor, while the dynamic range of PCG may be ranged between 5 and 2000 Hz. Despite this restriction, the custom device successfully recorded and measured the novel indices. Therefore, the next generation of PCG microphones (transducer) should be improved to closely reflect the actual dynamic range. In the future, the developed device can utilize a single printed circuit board that can further reduce its size. The ECG/PCG signals can be transferred wirelessly for fast recording and analysis, improving the turnaround time for clinical consultation. Moreover, this improved system can allow fast, convenient, and portable analysis of CTI parameters as well as novel indices that can be developed as gold standard tools in assessing myocardial function.

As expected, echocardiographic measurements of cardiac dimensions, function, and time intervals were within normal limits and revealed the healthy status of these dogs [26, 40, 45]. In addition, the cardiac biomarkers confirmed that these dogs were healthy, as all biomarker values were lower or within normal limits [46–48].

The ECG and PCG parameters of our device were comparable with the commercially available Eko DUO and the STE. Hence, this study was able to validate the accuracy of the device in describing ECG and PCG information in dogs. Recently, with the advent of newer technologies, a portable device that can simultaneously record heart sound and ECG became av­ailable. However, this product requires proprietary software and algorithms that are often calibrated for human use. Thus, we are interested in developing a novel device for dogs that can measure simultaneous ECG and PCG recordings without cost and proprietary limitations. By combining ECG and PCG recordings, we were able to measure cardiac timings and intervals that reflect the electrical and mechanical properties of the heart. These CTIs can provide information on the simultaneous cardiac events within the cardiac cycle and can be used to detect cardiac dysfunction. The ECG parameters in this study were comparable with previously reported values in conscious healthy Beagles [49, 50], while PCG parameters were similar to published reports in healthy dogs [51]. In the previous studies, it was reported that heart sound is correlated with the left ventricular systolic blood pressure and cardiac output [52, 53]. The use of digital PCG allows a visual and measurable description of heart sounds and murmurs in dogs that can be valuable in disease diagnosis [51, 54–56]. By adding together ECG and PCG signals, the characterization of arrhythmia and murmur can provide important information on cardiac function.

We established CTI values in healthy dogs using ECG and PCG recordings from our device and the Eko Duo device, which can be used as a baseline against CTI values in dogs with cardiac disease and can be compared in the future. The interplay of electrical and mechanical properties influences the volume, pressure, and blood flow within each heartbeat [1]. In the presence of diseases, these cardiac properties can be impaired [21, 57, 58]. These changes affect the systolic [59] and diastolic [60] functions of the heart and their related CTI parameters. The use of these parameters can be valuable as they can be used to screen different cardiac diseases [3–5, 30].

We compared systolic (QS1, QS2, and SPI), diastolic (S2S1), and global (MPI-F1, MPI-F2, and MPI-F3) time intervals between our custom device and the Eko device. These parameters signify electromechanical activation (QS1), electromechanical systole (QS2), and left ventricular systole (S1S2) [3, 29, 30, 61]. In this study, we were able to demonstrate that systolic parameters (QS1, QS2, and SPI) between these devices were comparable with minimal bias. In humans, STI derived from both PCG and seismocardiography were comparable with that of tissue Doppler imaging [35]. These parameters were also correlated with LVET [31] and are associated with left ventricular dysfunction. In addition, it can be used to identify heart failure patients [61]. Meanwhile, QS1 and QS2 in animals were both seen to correlate with invasive parameters of pressure-volume loop analysis [3]. In this study, the QS1 value (65.66 ± 7.61 ms) was comparably higher than the one reported in healthy Cavalier King Charles Spaniel dogs (33.4, SD 6.9) using separate PCG and ECG equipment and a 100 Hz filter [51]. The QS2 value from the device (299.50 ± 17.89 ms) is comparable with the values reported in the literature [6]. On the other hand, reference time points of SPI (QS1/S1S2) in healthy dogs using simultaneous ECG and PCG were not seen in the literature. Interestingly, the S1/S2 was seen as highly correlated with systolic blood pressure and was hypothesized to be related to myocardial contractile force [62], possibly, indicating systolic function. The clinical use of these STIs may be relevant in the presence of cardiac diseases such as MMVD, DCM, and advanced stages of heart failure where systolic dysfunction is apparent. In studies involving dogs with MMVD, significant shortening of S1S2 and S1/S2 was seen in higher stages of the disease, especially with severe left atrial and ventricular enlargement [51].

In addition to reflecting the heart sound, the diastolic time intervals provide a description of ventricular diastolic time (S2S1) [3, 28]. This diastolic time parameter did not differ from our device. The S2S1 in this study was comparable to the diastolic filling time as reported by Lavine *et al*. [63] in mixed breed dogs. In humans, the use of early diastolic time intervals can be useful in recognizing hypertrophic cardiomyopathy from hypertension [64] as well as DCM from ischemic cardiomyopathy [65]. In dogs, this diastolic filling time can be influenced by the size of the left ventricle, affecting vortex strength [66]. Several factors, such as rate of relaxation and atrial pressure can influence diastolic filling time [67]. In the presence of cardiac diseases, the effect of these factors can be further aggravated.

In this study, we proposed the formulas based on Wigger’s diagram and calculated for SPI and MPI based on simultaneous ECG and PCG recordings. We were able to demonstrate that these calculated formulas of SPI and MPI did not differ and were in close agreement between Eko DUO and our device. The previous studies in humans concerning parameters from acoustic cardiography revealed its association with invasive measurement of the left ventricular contractility and its ability to recognize heart failure phenotypes [2, 68, 69].

The use of echocardiography is considered the gold standard tool to describe abnormal changes and functions of the heart [21, 70, 71]. However, accessibility, cost, and technical expertise are constraining factors in its use in general practice. As a result, we are looking for methods that may be used clinically to describe heart function. Hence, we further validated the usefulness of SPI and MPI formulas derived from CTI parameters. We compared them with analogous CTI parameters from STE. The PEP/LVET was comparable with SPI from our device while MPI-F2 and MPI-F3 were equivalent to the Tei index from STE. The use of acoustic cardiography was shown to be compared with the pulsed-wave Doppler echocardiography in cardiac resynchronization therapy in humans but with ease of use [72]. We did not notice a similar study on healthy dogs. However, we previously reported the use of similar CTI parameters in MMVD dogs [37]. This study found that MPI-F1 may not be interchangeably used with the Tei index as it demonstrated a wide disagreement. In addition, when compared to the previous reports, the calculated MPI from the device was higher than the observed values using Doppler transthoracic echocardiography in conscious [16] and anesthetized [5] healthy dogs. These differences may be influenced by several factors, including environmental stimuli, differences in the technical software of a machine, the use of an algorithm, and filters used for signal processing. For example, a higher sensitivity to retrieve heart sound signals by our device may amplify the heart sound recordings that influence the measurement and calculation. As a result, higher values may be obtained from the device compared to STE, which utilized its own algorithm.

We investigated further if the SPI and calculated MPI formulas as we proposed can reveal a significant association with echocardiographic parameters. We did not see any significant association between SPI, F1, F2, and F3 with cardiac dimensions, cardiac function, STI, and global cardiac function parameters from STE. In our study, we only found a significant correlation between SPI and MCOdur, as well as MPI-F3 and HR. These findings may have been influenced by sympathetic stimuli affecting HR and consequently calculated SPI and MPI-F3. The MPI is not influenced by HR [73–75] and these findings were similarly observed in SPI, MPI-F1, and MPI-F2 in this study. However, changes in volume load can affect MPI [75], particularly in cases of cardiac dysfunction. Therefore, these parameters may be useful in cases of cardiac diseases where systolic, diastolic, and global cardiac dysfunctions are evident. These cardiac dysfunctions may subsequently alter SPI and MPI parameters that can be detected by the device.

## Limitations

This study utilized a limited number of healthy dogs. However, a homologous population in terms of age and breed, the establishment of reference CTI values in healthy dogs may be better validated using a larger number of populations. Although CTI parameters measured by the device were repeatable with a low standard error of the mean, we did not measure interobserver variability. The use of 2–3 observers and the use of inter and intra-operability tests can strengthen further the clinical use of these proposed parameters and will be good components of our future research. The use of conscious, young, active, and healthy dogs in determining CTI values may be influenced by external and sympathetic stimuli. Finally, the variation in the filter, signal processing, and recording software between these devices should be noted. The role of these CTIs and the calculated MPI formulas can be further investigated in the presence of cardiac diseases in dogs, including MMVD and DCM where cardiac dysfunction is evident.

## Conclusion

The custom device based on simultaneous ECG and PCG recordings was developed and validated its use. CTI parameters were measured and established in healthy beagles as baseline recording from this device. In addition, these CTIs were comparable to those obtained from the commercially available device or echocardiography. Furthermore, we proposed formulas derived from CTI parameters that seemed analogous to the systolic function parameter and global cardiac function parameters from Doppler echocardiography. The establishment of these CTI values can be helpful in clinical practice to initially describe cardiac function during a routine examination. This tool was seen to be accurate, cheap, comparable, and convenient, in particular in the practice where access to STE is inadequate or limited.

## Authors’ Contributions

KRBG, CP, KK, SS, SK, SA, PN, and KT: Data collection. KRBG, KK, WS, PN, and KT: Writing. KT: Project administration. KK and PN: Development of device. All authors have read and approved the final manuscript.
